# Effect of nanoparticle size on their distribution and retention in chronic inflammation sites

**DOI:** 10.1186/s11671-023-03882-w

**Published:** 2023-08-22

**Authors:** Abdulaziz M. Aldayel, Stephanie Hufnagel, Hannah L. O’Mary, Solange A. Valdes, Riyad F. Alzhrani, Haiyue Xu, Zhengrong Cui

**Affiliations:** 1 College of Pharmacy, Division of Molecular Pharmaceutics and Drug Delivery, The University of Texas at Austin, Austin, TX 78712 USA; 2 Nanomedicine Department, King Abdullah International Medical Research Center (KAIMRC), King Abdulaziz Medical City (KAMC), 11426 Riyadh, Saudi Arabia; 3 King Saud Bin Abdulaziz University for Health Sciences (KSAU-HS), King Abdulaziz Medical City (KAMC), 11426 Riyadh, Saudi Arabia; 4 Department of Pharmaceutics, College of Pharmacy, King Saud University, 11451 Riyadh, Saudi Arabia

**Keywords:** Nanoparticles, Chronic inflammation, Size-dependent, Retention, Specificity

## Abstract

Nanomedicines are increasingly researched and used for the treatment of chronic inflammatory diseases. Herein, the effect of the size of nanoparticles on their distribution and retention in chronic inflammatory sites, as compared to healthy tissues, was studied in a mouse model with chronic inflammation in one of the hind footpads. Using PEGylated gold nanoparticles of 2, 10, 100, and 200 nm, we found that although the smaller nanoparticles of 2 and 10 nm showed greater distribution and slower clearance in the inflamed footpad than the relatively larger nanoparticles of 100 and 200 nm, the larger nanoparticles of 100 and 200 nm were more selectively distributed in the inflamed hind footpad than in the healthy hind footpad in the same mouse. Based on these findings, we prepared protein nanoparticles of 100–200 nm with albumin, IgG antibody, or anti-TNF-α monoclonal antibody (mAb). The nanoparticles can release proteins in response to high redox activity and/or low pH, conditions seen in chronic inflammation sites. We then showed that upon intravenous injection, those stimuli-responsive protein nanoparticles distributed more selectively in the inflamed footpad than free proteins and remained longer in the inflamed footpad than similar protein nanoparticles that are not sensitive to high redox activity or low pH. These findings support the feasibility of increasing the selectivity of nanomedicines and protein therapeutics to chronic inflammation sites and prolonging their retention at the sites by innovative nanoparticle engineering.

**Graphical Abstract:**

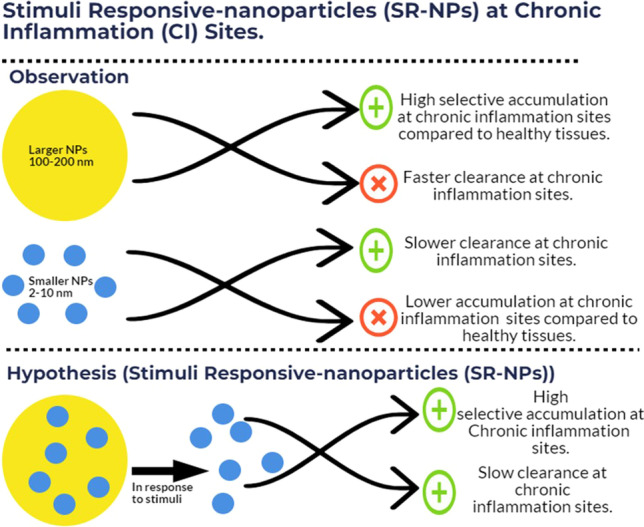

**Supplementary Information:**

The online version contains supplementary material available at 10.1186/s11671-023-03882-w.

## Introduction

About 50% of all health-related deaths are linked to inflammation-related diseases such as cancer and heart diseases [1–3]. Nanoparticles have been extensively studied to increase the efficacy and/or reduce the toxicities of drugs in several diseases such as cancer and inflammation [1–15]. Nanoparticle parameters such as size and surface charge have been shown to affect their biodistribution and clearance, and thereby the efficacy of nanoparticle-based therapies [5, 8, 16–25]. For example, it is known that nanoparticle size has a significant effect on their distribution and accumulation in tumor tissues and chronic inflammation sties [26, 27]. The chronic inflammation sites in many diseases such as rheumatoid arthritis have a demonstrated phenomenon known as the extravasation through leaky vasculature and subsequent inflammatory cell-mediated sequestration (ELVIS) [28, 29]. Several nanoparticle systems (e.g., gold nanoparticles, albumin nanoparticles, lipid and polymeric nanoparticles, and biomimetic nanoparticles) have shown greater accumulation at inflammation sites, presumably due to the ELVIS effect [30]. Unlike the enhanced permeability and retention (EPR) effect seen in solid tumor tissues, the lymphatic system in chronic inflammation sites is active via inflammatory cell-mediated clearance [28–32]. However, the effect of the size of nanoparticles on their distribution and retention in chronic inflammation sites is not well studied, which is critical considering that monoclonal antibodies (mAbs) such as anti-TNF-α mAbs, which are physically nanoparticles, are clinically used in treating chronic inflammatory diseases [33–36].

In the present study, we first investigated the size-dependent distribution and retention of PEGylated gold nanoparticles of different sizes (i.e., 2, 10, 100 and 200 nm) in chronic inflammation sites in a mouse model with lipopolysaccharide (LPS)-induced chronic inflammation. Unlike other chronic inflammation models such as collagen-induced arthritis, the LPS-induced chronic inflammation can be limited to one foot in a mouse, at least initially, thereby allowing the testing of the specificity of the nanoparticles toward the inflamed foot as compared to a healthy non-inflamed foot in the same mouse [5–7, 37–41]. We then confirmed the finding using redox-sensitive nanoparticles engineered by crosslinking proteins (i.e., albumin, IgG, anti-TNF-α mAb) with disulfide bonds and showed that the resultant nanoparticles not only increased the distribution of the proteins in chronic inflammation sites, but also increased their retention in the sites.

## Materials and methods

### Materials

Lugol's solution, Tris–EDTA (TE), sodium dodecyl sulfate (SDS), Triton X-100, N,N-dimethyl-9,9-biacridinium dinitrate (lucigenin), LPS from *Salmonella enterica* serotype enteritidis, 3-aminophthalhydrazide, 5-amino-2,3-dihydro-1,4-phthalazinedione (luminol sodium salt), bovine serum albumin (BSA) (lyophilized powder, ≥ 96%), 3,3'-dithiobis(sulfosuccinimidyl propionate) (DTSSP), glutathione, and sulfosuccinimidyl 4-(N-maleimidomethyl)cyclohexane-1-carboxylate (sulfo-SMCC) were from Sigma-Aldrich (St. Louis, MO) [38]. Albumin-Alexa Fluor™ 647 conjugate and albumin from bovine serum (BSA)-FITC conjugates were from ThermoFisher (Waltham, MA). Normal mouse IgG Alexa Fluor® 647 was from Santa Cruz Biotechnology (Dallas, TX) [38]. InVivoMAb anti-mouse TNF-α (anti-TNF-α mAb) was from BioXCell (West Lebanon, NH), and mouse TNF-α ELISA MAX™ Standard was from Biolegend (San Diego, CA). Dulbecco's Modified Eagle Medium (DMEM), fetal bovine serum (FBS), and streptomycin/penicillin were from Invitrogen (Carlsbad, CA). The 2-mercaptoethanol was from Bio-Rad (Hercules, CA).

### Gold nanoparticles

PEGylated gold nanoparticles of the following sizes, 2, 10, 100, and 200 nm, labeled with Cy7.5, were from NANOCS (New York, NY). The nanoparticles have a uniform size distribution measured by dynamic light scattering (DLS) and transmission electron microscopy (TEM) by the manufacturer. In addition, we confirmed the size of the 10 and 100 nm DLS and TEM. Hydrodynamic size and zeta potential were measured using a Malvern ZetaSizer ZS (Westborough, MA). For TEM, nanoparticles were deposited onto copper grids, stained with phosphotungstic acid (PTA) (2% w/v), and dried overnight [38]. For more information about nanoparticle synthesis and characterization please visit Nanocs (http://www.nanocs.net/nanoparticle/gold-nanoparticles.htm). The fluorescence intensity of nanoparticles was measured using a Spectrum IVIS (Caliper, Hopkinton, MA), and nanoparticles that showed a higher fluorescence intensity were diluted with phosphate buffered saline (PBS, 10 mM, pH 7.4) so that all the nanoparticles in suspension had a similar fluorescence intensity before they were injected into animals [37–39, 42]. PEG content on the surface of the nanoparticles was measured using an iodide staining method with Lugol's solution. Briefly, 150 µl of (7 × 10^12^ nanoparticles/ml) were added to a solution that contained 950 μl of PBS (pH 7.4, 10 mM) and 68 μl of Lugol's solution [38]. After 5 min of incubation at room temperature, the absorbance (OD490 nm) was measured using a BioTek Synergy Microplate Reader (Winooski, VT).

### Preparation and characterization of stimulus responsive (SR)-albumin nanoparticles

BSA was used to formulate the SR-albumin nanoparticles via a desolvation technique as previously described [42]. BSA was dissolved at a concentration of 25 mg/ml in a 10 mM sodium phosphate solution (pH 9.0). The solution was filtered through a 0.22 μm filtration unit (Schleicher und Schüll, Dassel, Germany). An Aliquot (1.0 ml) of the BSA solution was transformed into nanoparticles by dropwise addition of 4.0 ml of the desolvating agent (i.e., ethanol/methanol, 50/50%) under stirring at room temperature [38]. After the desolvation process, 100 μl of 1% DTSSP in water solution was added to induce crosslinking. The crosslinking process was performed under stirring over 24 h at room temperature [38]. Similarly, stable albumin nanoparticles (i.e., stimulus non-responsive (SnR)-albumin nanoparticles) were prepared using 100 μl of a 1% sulfo-SMCC solution as the crosslinker [38]. For animal studies, 5 mg of the albumin-Alexa Fluor™ 647 conjugate was added to 20 mg of BSA to prepare fluorescently labeled SR-albumin nanoparticles and SnR-albumin nanoparticles [38]. For the uptake study, 1 mg of the albumin-FITC conjugate was added to 24 mg of BSA to fluorescently label the SR-albumin nanoparticles [38].

The particle size, polydispersity index (PDI), and zeta potential of the nanoparticles were determined using a Malvern Zeta Sizer Nano ZS. For the release study, 1% of 2-mercaptoethanol was prepared in PBS (10 mM, pH 7.4) to test the stability of SR-albumin nanoparticles in high redox conditions [38]. SR-albumin nanoparticles or SnR-albumin nanoparticles were collected by centrifugation (17,500 × *g*, 30 min, 4 °C), resuspended in 1 ml of 1% of 2-mercaptoethanol in PBS or PBS alone (10 mM, pH 7.4), and then placed in shaker incubator (MAQ 5000, MODEL 4350, Thermo Fisher Scientific, Waltham, MA) (100 rpm, 37 °C). In another experiment, SR-albumin nanoparticles were placed in PBS (10 mM, pH 6.8 or pH 7.4) to study the effect of pH on the stability of the nanoparticles [38]. After 2 h, the tubes were centrifuged (17,500 × *g*, 30 min), and the amount of albumin released (i.e., in the supernatant) was measured using a Bradford assay by measuring the absorbance at 595 nm using a microplate reader [38].

### Preparation and characterization of SR-IgG-NPs

Normal mouse IgG lableled with Alexa Fluor® 647 was used to prepare the SR-IgG-NPs via the desolvation technique as previously described [38]. IgG was diluted to a concentration of 500 µg/ml in 10 mM sodium phosphate solution (pH 9.0). The resulting solution was filtered through a 0.22 μm filtration unit. Aliquot (1.0 ml) of the IgG solution was transformed into nanoparticles by dropwise addition of 4.0 ml of the desolvating agent (i.e., ethanol/methanol, 50/50%) under stirring (500 rpm) at room temperature [38]. After the desolvation process, 100 μl of 0.04% DTSSP in water was added to induce crosslinking. The crosslinking process was performed under stirring over a time period of 24 h at room temperature [38]. The particle size, PDI, and zeta potential of the nanoparticles were determined. For the release experiment, the SR-IgG nanoparticles were placed in PBS (10 mM, pH 7.4) or 1% of 2-mercaptoethanol in PBS to study the effect of a high redox condition on the particle stability [38]. After 2 h, the tubes were centrifuged (17,500 × g, 30 min), and the amount of IgG released (i.e., in the supernatant) was measured using a Bradford assay [38].

### Preparation and characterization of SR-TNF-α mAb nanoparticles

InVivoMAb anti-mouse TNFα (anti-TNF-α mAb) was used to prepare the SR-TNF-α mAb-NPs [38]. Briefly, the TNF-α mAb was diluted to a concentration of 1 mg/ml in a 10 mM sodium phosphate solution (pH 9.0). Aliquot (1.0 ml) of the anti-TNF-α mAb solution was transformed into nanoparticles by dropwise addition of 4.0 ml of a desolvating agent (i.e., ethanol/methanol, 50%/50%) under stirring (500 rpm) at room temperature [38]. After the desolvation process, 100 μl DTSSP in water solution (0.04%) was added to induce crosslinking (i.e., 24 h at room temperature under stirring). The particle size, PDI, and zeta potential of the nanoparticles were determined [38].

### TEM and scanning transmission electron microscopy (STEM)

Morphological characterization of the nanoparticles was performed using TEM. One drop of nanoparticle suspension (5 μl) was applied to carbon-coated 400 mesh grids after activation and allowed to dry prior to examination [38]. Nanoparticles were visualized using an FEI Tecnai Transmission Electron Microscope (Hillsboro, OR). For STEM, carbon film-coated copper grids (Electron Microscopy Sciences (EMS), Hatfield, PA) were first processed with an EMS Sputter Coater. Next, approximately 5 µl of the aqueous sample of nanoparticles was placed onto the grid and the sample was allowed to air-dry before examination using a Hitachi S-5500 SEM/STEM (Hitachi, Santa Clara, CA) available at UT Austin [38].

### In vitro cellular uptake of BSA and SR-albumin nanoparticles

Murine macrophage J774A.1 cells (American Type Culture Collection, Manassas, VA) were seeded in a 12-well plate (2 × 10^5^ cells/well). Free BSA-FITC conjugate or fluorescently labeled SR-albumin nanoparticles were added to the cell culture medium [38]. After 50 min of co-incubation, the cells were washed with PBS (10 mM, pH 7.4) and lysed with a lysis solution that contained 2% (v/v) SDS and 1% Triton X-100 [38]. The fluorescence intensity in the cell lysate was measured using a microplate reader (Ex = 485 nm, Em = 528 nm). A Bradford protein assay did not show any significant difference in the total protein concentrations in the lysates between the groups [38].

### Testing of the binding activity of the anti-TNF-a mAbs released from SR-TNF-α mAb nanoparticles

The nanoparticles were centrifuged at 15,000 rpm for 30 min, and the pellet was suspended in 1 ml of pH 6.8 and 0.015% β-mercaptoethanol or PBS pH 7.4, which was then placed in shaker incubator (MAQ 5000, MODEL 4350) for about 1.5 h (150 rpm, 37 °C) [38]. To determine the ability of TNF-α mAb released to bind to mouse TNF-α, 12.5 μg (0.5 ml of 25 μg added to 0.5 ml of the samples) of free TNF-α mAb or SR-TNF-α mAb nanoparticles were incubated with 62.5 pg/ml of mouse TNF-α protein in a shaker incubator for 2 h (150 rpm, 37 °C). The TNF-α concentrations in the samples were measured using a Mouse TNF-α ELISA MAX™ Standard from Biolegend.

### Induction of chronic inflammation in mice using LPS

The animal protocol was approved by the Institutional Animal Care and Use Committee at The University of Texas at Austin. Female C57BL/6 mice (6–8 weeks) were from Charles River Laboratories (Wilmington, MA). For imaging, mice were fed with alfalfa-free diet (Harlan, Indiana) to minimize unwanted background signals [37–39, 42]. LPS-induced mouse model of chronic inflammation was established as previously described [37–39]. Briefly, LPS was dissolved in sterile PBS (pH 7.4, 10 mM) at a concentration of 1 mg/ml. A 50 μl of the solution was injected into the right hind footpad of each mouse on day 0 [37–39, 42]. Acute inflammation was confirmed using a Spectrum IVIS® with a bioluminescence imaging system 20 min following intraperitoneal (i.p.) injection of luminol (100 mg/kg) (exposure time 60 s, large binning, field B) on day 3 [37–39, 42]. Chronic inflammation was confirmed on day 8 using a Spectrum IVIS® with a bioluminescence imaging system 20 min following i.p. injection of lucigenin (15 mg/kg) (exposure time 60 s, large binning, field B). Only mice that showed significant chronic inflammation in the right foot were used for additional experiments.

### Pharmacokinetics (PK) and distribution of nanoparticles in chronic inflammation sites in a mouse model

Upon the confirmation of chronic inflammation in the right rear footpad of mice, mice were randomized into groups and injected intravenously (i.v.) with PBS or gold nanoparticles of different particle sizes with same fluorescence intensity (adjusted by dilution) [37–39, 42]. Mice were imaged using a Spectrum IVIS at different time points after the injection (i.e., 3, 6, 12, and 24 h and 2, 4, 8, and 16 days). At the end of the study, mice were euthanized to collect the inflamed foot and major organs (i.e., heart, kidneys, liver, spleen, and lung). All samples were weighed and imaged using a Spectrum IVIS. All fluorescence units are in photons per second per centimeter square per steradian (p/s/cm^2^/sr).

Similarly, groups of mice with confirmed chronic inflammation in the right rear footpad were i.v. injected with PBS, free albumin, SnR-albumin-NPs, or SR-albumin-NPs (albumin-Alexa Fluor™ 647, 0.32 mg/kg). Mice were imaged using a Spectrum IVIS at various time points after the injection (i.e., 3, 6, 12, 24 h and 2, 4, 6, and 7 days). Another study was also carried out using fluorescently labeled SR-IgG-nanoparticles (IgG, 2 µg/kg). The fluorescent intensity was measured and adjusted to the same for all formulations, except for PBS, before i.v. injection [37–39, 42]. Mice were imaged using a Spectrum IVIS at various time points (i.e., 3, 6, 12, 24 h and 2, and 4 days) after the injection. All data were analyzed using PK Solver to derive PK parameters [43].

### Statistical analyses

Statistical analyses were completed by performing an analysis of variance followed by Fisher’s protected least significant difference procedure. A p-value of ≤ 0.05 (two-tail) was considered significant.

## Results and discussion

### Effect of the size of gold nanoparticles on their PK and distribution in inflammation sites

**Fig. 1:**
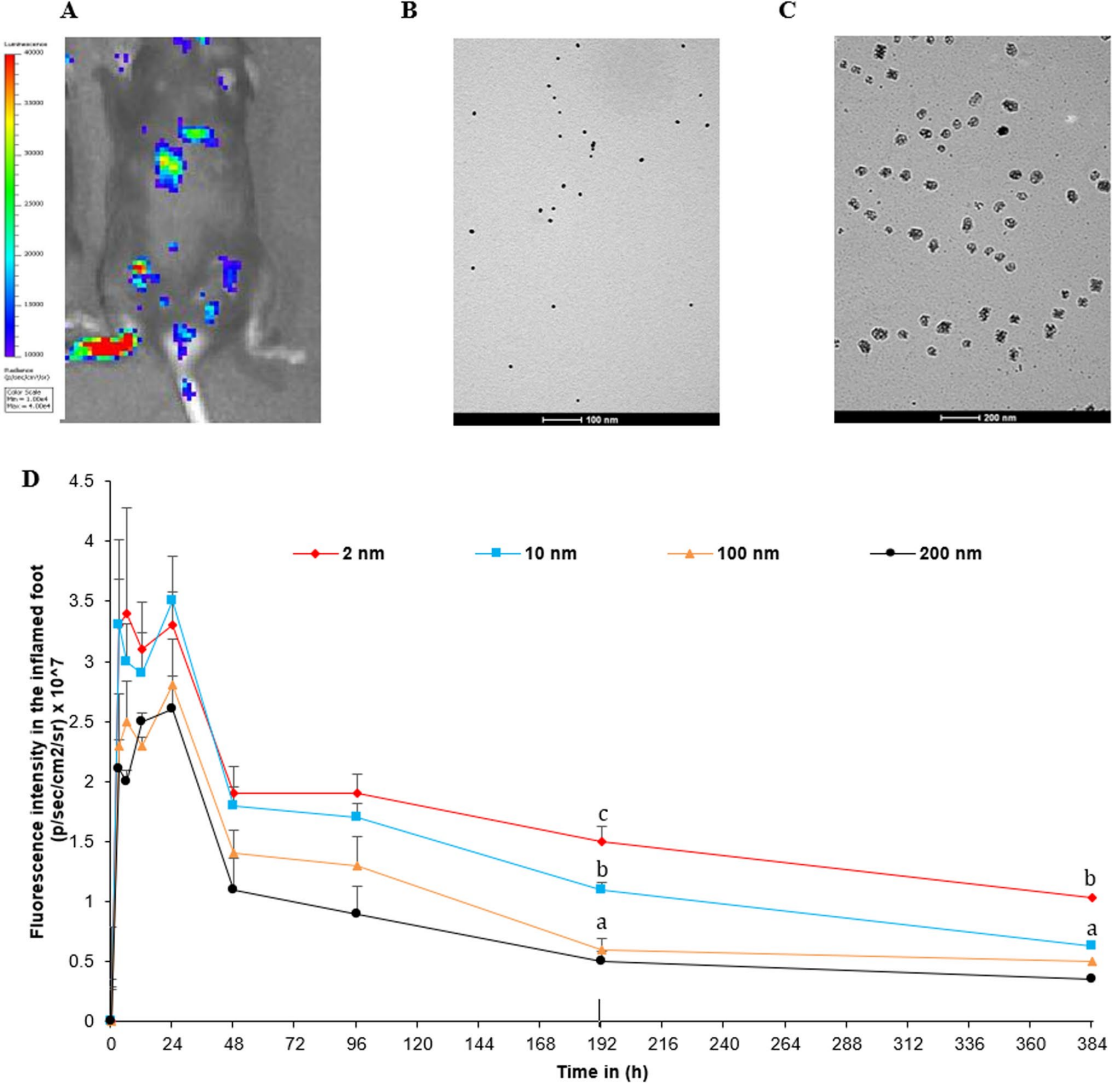
PK of 2 nm, 10 nm, 100 nm, and 200 nm fluorescently labeled gold nanoparticles in the inflamed foot in mice. **A** In vivo longitudinal bioluminescence imaging of chronic inflammation of the right foot pad. Local inflammation was induced by s.c. injection of 50 μg LPS in the hind footpad, and mice were given an i.p. injection of lucigenin (25 mg/kg, i.p.) on day 8. **B** Representative TEM images of the 10 nm PEGylated gold nanoparticles. **C** Representative TEM images of the 100 nm PEGylated gold nanoparticles. (D) Shown are in vivo fluorescence intensity-time profiles. Data are mean ± S.E. (n = 3–5). (a-c, *p* < 0.05)

The well-established mouse model of LPS-induced chronic inflammation can be used to test the effect of the size of the nanoparticles on their distribution and retention in chronic inflammation sites, relative to healthy, non-inflammatory sites [5–7, 37–41]. After the LPS was injected into the footpad of one of the hind legs of a mouse, acute inflammation developed 2–3 days later, and chronic inflammation became apparent around days 8–9 in the injected footpad, but not in the footpad of the other hind leg (Fig. 1A), which allows us to evaluate the distribution of the fluorescently labeled nanoparticles in the inflamed footpad, relative to the footpad without chronic inflammation, in the same mouse using in vivo fluorescence imaging.

Mice with confirmed chronic inflammation in the right hind footpad were grouped and i.v. injected with fluorescently-labeled, PEGylated gold nanoparticles of various sizes. Representative TEM images of the 10 and 100 nm gold nanoparticles are shown in Fig. 1B and Fig. 1C, respectively, and the PEGylation of the nanoparticles was confirmed by estimating PEG content with an iodide staining method (data not shown). It is noted that the PEGylated gold nanoparticles are not spherical. Shown in Fig. 1D are the fluorescence intensity vs. time curves in the inflamed footpad of mice i.v. injected with gold nanoparticles of different sizes. Selected PK parameters are shown in Table 1 and Supp Table 1. Overall, the area under the curve (AUC) values of the gold nanoparticles of 2 nm and 10 nm in diameters were greater than those of the gold nanoparticles of 100 and 200 nm in diameter (Fig. 1D, Table 1), and the small 2 nm and 10 nm gold nanoparticles showed a relatively slower clearance than the 100 and 200 nm gold nanoparticles. Finally, all the gold nanoparticles reached their maximum concentrations in the inflamed footpad within 24 h of injection (Fig. 1D).

**Table 1:** PK parameters of 2 nm, 10 nm, 100 nm and 200 nm fluorescently labeled gold nanoparticles in the inflamed foot in mice. selected pharmacokinetic parameters. (n = 3–5)

Inflamed foot PK parameter	2 nm	10 nm	100 nm	200 nm
AUC_0-t (fluor/ml*h)_	632.6	518.6	367.7	294.6
t_1/2 (h)_	329.8	213.3	214.6	203
Cl/F_obs _(fluor)/(fluor/ml)_	0.019	0.031	0.042	0.055
C_max (fluor/ml)_	3.4	3.5	2.8	2.6

**Fig. 2:**
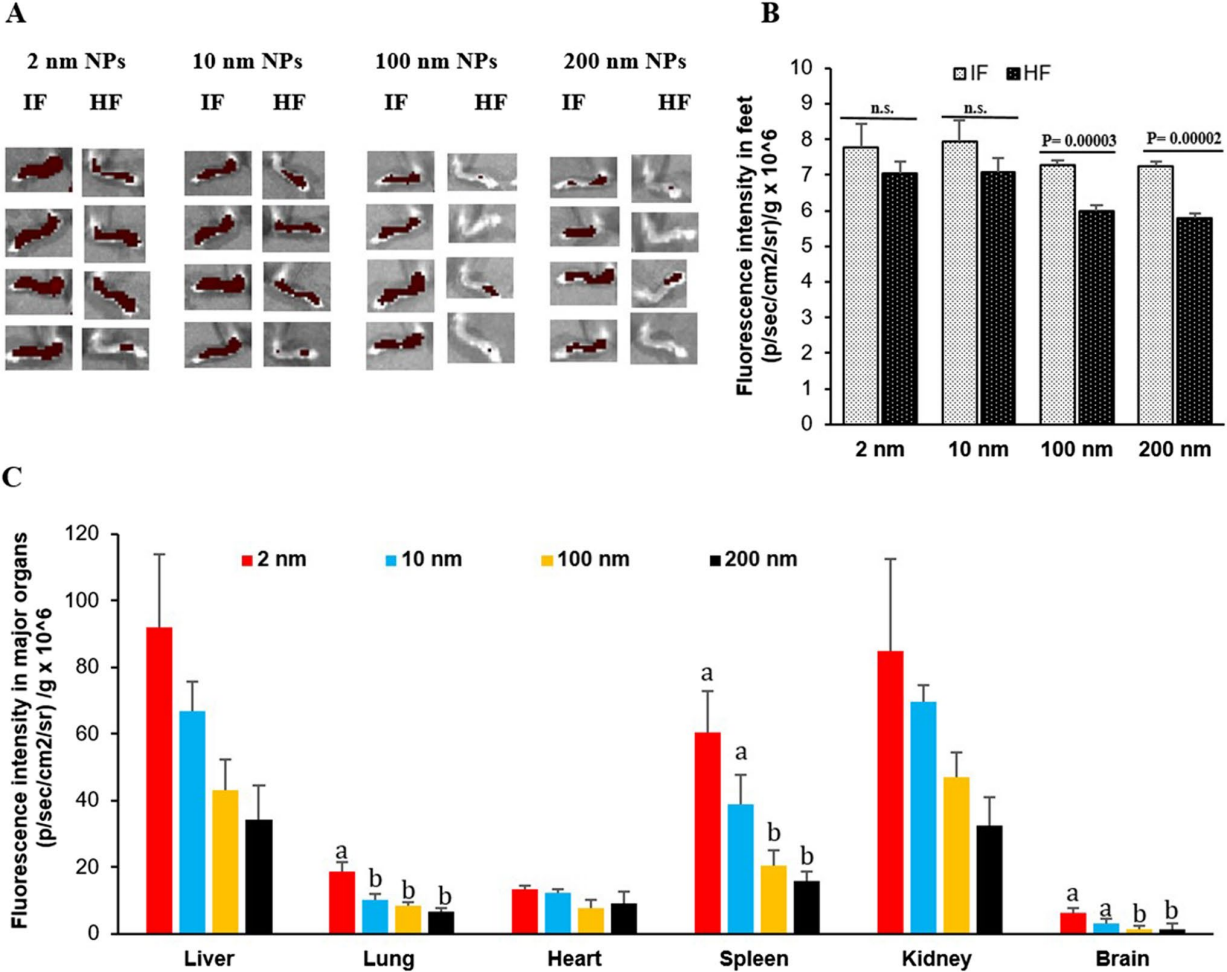
Selective distribution of 2 nm, 10 nm, 100 nm, and 200 nm fluorescent nanoparticles towards inflamed foot relative to healthy foot and in major organs. **A** In vivo fluorescence images of inflamed mouse feet vs. healthy feet at 24 h after i.v. injection of 2 nm, 10 nm, 100 nm, and 200 nm nanoparticles. IF = Inflamed Foot., HF = Healthy Foot. **B** Fluorescence intensity values of healthy vs. inflamed foot of the 2 nm, 10 nm, 100 nm, and 200 nm treated mice at 24 h post i.v. injection. **C** Normalized fluorescence intensity values in major organs of mice 16 days after they were i.v. injected with the gold nanoparticles. The significance of the groups is relative to the 2 nm group. The gold nanoparticles were PEGylated and labeled with Cy 7.5. Data are mean ± S.D. (n = 4). (*p < 0.05) or (a-c, *p* < 0.05)

Shown in Fig. 2A are the IVIS images of hind legs from the same mouse, one with chronic inflammation and the other not, 24 h after the mouse was i.v. injected with gold nanoparticles of 2, 10, 100, or 200 nm. In mice i.v. injected with the gold nanoparticles of 100 nm or 200 nm, the fluorescence intensities in the inflamed footpad were higher than in the healthy footpad (*p* = 0.0003 and 0.0002, respectively). In contrast, in mice i.v. injected with the 2 nm or 10 nm gold nanoparticles, the fluorescence intensities in the inflamed footpad were not different from that in the healthy footpad (Fig. 2A). Figure 2B shows the ratios of the fluorescence intensities in the inflamed footpad vs. in the healthy footpad for the gold nanoparticles of various sizes. For the 100 nm and 200 nm gold nanoparticles, the ratios were higher than 20, and for the 2 nm and 10 nm gold nanoparticles, their ratios were in the range of 10–15 (Fig. 2B), indicating that the distribution of the relatively larger 100 and 200 nm gold nanoparticles to the inflamed footpad was more selective than the distribution of the smaller 2 and 10 nm gold nanoparticles, although more smaller gold nanoparticles of 2 nm and 10 nm reached the footpads, inflamed or not (Figs. 1D, 2A, and Table 1).

Finally, upon euthanization of the mice, key organs including the liver, heart, spleen, lung, kidneys, and brain were harvested and weighed, and the fluorescence intensity values were measured. As shown in Fig. 2C, the 100 and 200 nm gold nanoparticles, especially the 200 nm gold nanoparticles, had a relatively lower distribution to those organs, as compared to the smaller 2 or 10 nm gold nanoparticles. This is in agreement with data reported by Hirn et al. (2011) using negatively charged, spherical gold nanoparticles of different particle sizes (i.e., 1.4, 5, 18, 80, and 200 nm) [18].

Our data in Figs. 1, 2 showed that the distributions of the smaller nanoparticles (i.e., 2 and 10 nm) in the inflamed footpad and the healthy footpad are not significantly different, but the distribution of the larger nanoparticles (i.e., 100 and 200 nm) in the inflamed foot was rather selective and significantly higher than in the healthy foot. The term of ELVIS was previously coined to explain the passive accumulation of large molecules and nanoparticles in chronic inflammation sites due to the disruption of gap junctions between endothelial cells at the inflammation sites [26]. Our data show that the size of the nanoparticles also significantly affects their distribution in the chronic inflammation site. Relatively smaller nanoparticles (e.g., 2 or 10 nm) have a significantly higher distribution and accumulation in the chronic inflammation site than relatively larger nanoparticles (e.g., 100–200 nm); however the relatively larger nanoparticles showed a higher selectivity in their distribution in chronic inflammation sites than in healthy tissues. The exact mechanism underlying these differences is unknown but it is likely related to the relative permeability of nanoparticles of different sizes through the blood capillary in chronic inflammation sites as well as the difference in the immune cells’ ability to take up nanoparticles of different sizes [28, 29]. Another possible explanation is the potential uptake by blood-circulating myeloid cells that ends up transporting the nanoparticles into the inflammation sites [28, 29].

### Nanoparticles of 100–200 nm that can dissociate/disintegrate in chronic inflammation sites have a higher selectivity to chronic inflammation sites than smaller nanoparticles

**Fig. 3:**
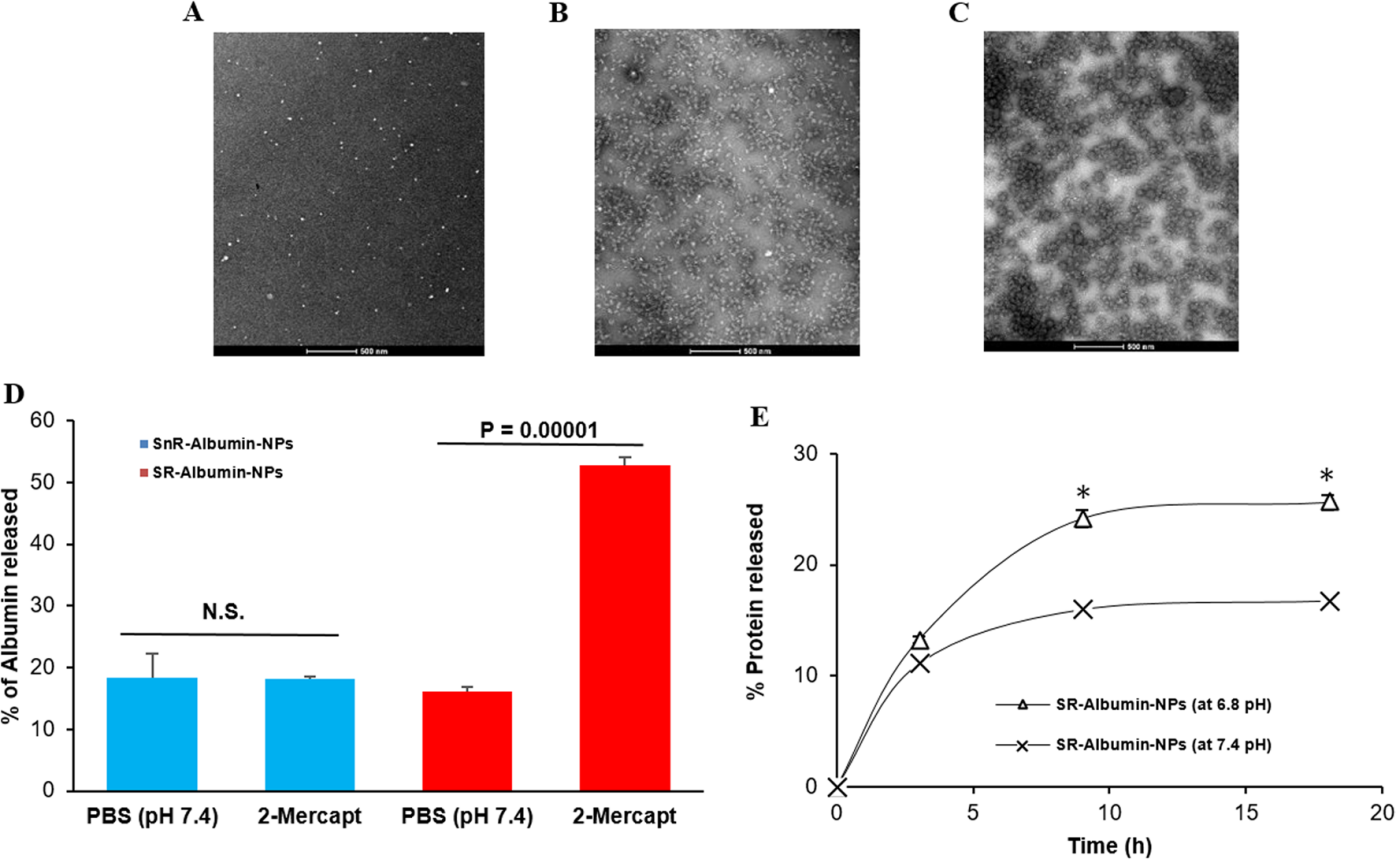
In vitro redox and pH-sensitivity of the SR-albumin nanoparticles. **A** Representative TEM images of the free albumin, **B** of the SnR-albumin-NPs, and **C** of the SR-albumin-NPs. **D** In vitro release of albumin from the SnR-albumin nanoparticles and the SR-albumin nanoparticles at 2 h after pre-incubation in PBS or 1% of 2-mercaptoethanol. **E** In vitro release of protein from the SR-albumin nanoparticles in pH 7.4 vs. pH 6.8 (chronic inflammation like microenvironment). Data are mean ± S.E. (n = 3). * p value ≤ 0.05

Based on the findings with the gold nanoparticles, it was hypothesized that nanoparticles of 100–200 nm that dissociate (or disintegrate) into small nanoparticles of 2–10 nm in chronic inflammation sites would have a more selective distribution to the chronic inflammation sites than small nanoparticles of 2–10 nm and an enhanced retention in the chronic inflammation sites than nanoparticles of 100–200 nm that do not dissociate (disintegrate) into small nanoparticles in chronic inflammation sites. To test this hypothesis, albumin was used as a representative of small nanoparticles of 2–10 nm and was crosslinked into larger nanoparticles of around 190 nm in diameter using disulfide bonds, which are redox- and pH-sensitive (i.e., SR-albumin nanoparticles) or using thioether bonds, which are not redox-sensitive (i.e., SnR-albumin nanoparticles). Shown in Table 2 are the particle size, zeta potential, and polydispersity index of the resultant nanoparticles. Representative TEM images of the albumin, SR-albumin nanoparticles, and SnR-albumin nanoparticles are shown in Fig. 3A–C, respectively. There is not any significant difference between the SR-albumin nanoparticles and the SnR-albumin nanoparticles in their morphology, size (~ 190 nm), and zeta potential values. However, due to the relatively high-redox activity and low pH in chronic inflammation sites [43–48], it is expected that the SR-albumin nanoparticles, unlike the SnR-albumin nanoparticles, will disintegrate and release albumin when they reach chronic inflammation sites. The redox-sensitivity of the SR-albumin nanoparticles was confirmed in vitro by incubating the nanoparticles with 2-mercaptoethanol to facilitate the cleavage of the disulfide bonds among the albumin proteins in the SR-albumin-nanoparticles and thus as expected accelerate the release of the albumin from the nanoparticles (Fig. 3D). In addition, the acid-sensitivity of the SR-albumin nanoparticles was also confirmed in vitro by incubating the nanoparticles at pH 6.8 versus at pH 7.4 (Fig. 3E). The faster release of albumin from the SR-albumin nanoparticles in response to both high redox activity and a relatively lower pH (i.e., 6.8, similar to that in chronic inflammation sites) is expected to be beneficial in increasing the retention of the albumin in the chronic inflammation sites, as the albumin (~ 66 kDa) are physically small nanoparticles.

**Table 2:** Characterization of stimulus responsive (SR)-albumin nanoparticles and stimulus non-responsive (SnR)-albumin nanoparticles. Data are mean ± S.E. (n = 3)

Final formulation	Particle size (nm)	PDI	Zeta potential (mV)
SnR-Albumin-NPs	187.3 ± 21	0.26 ± 0.02	− 26.0 ± 2.1
SR-Albumin-NPs	186.3 ± 12	0.27 ± 0.03	− 23.7 ± 2.0

**Fig. 4:**
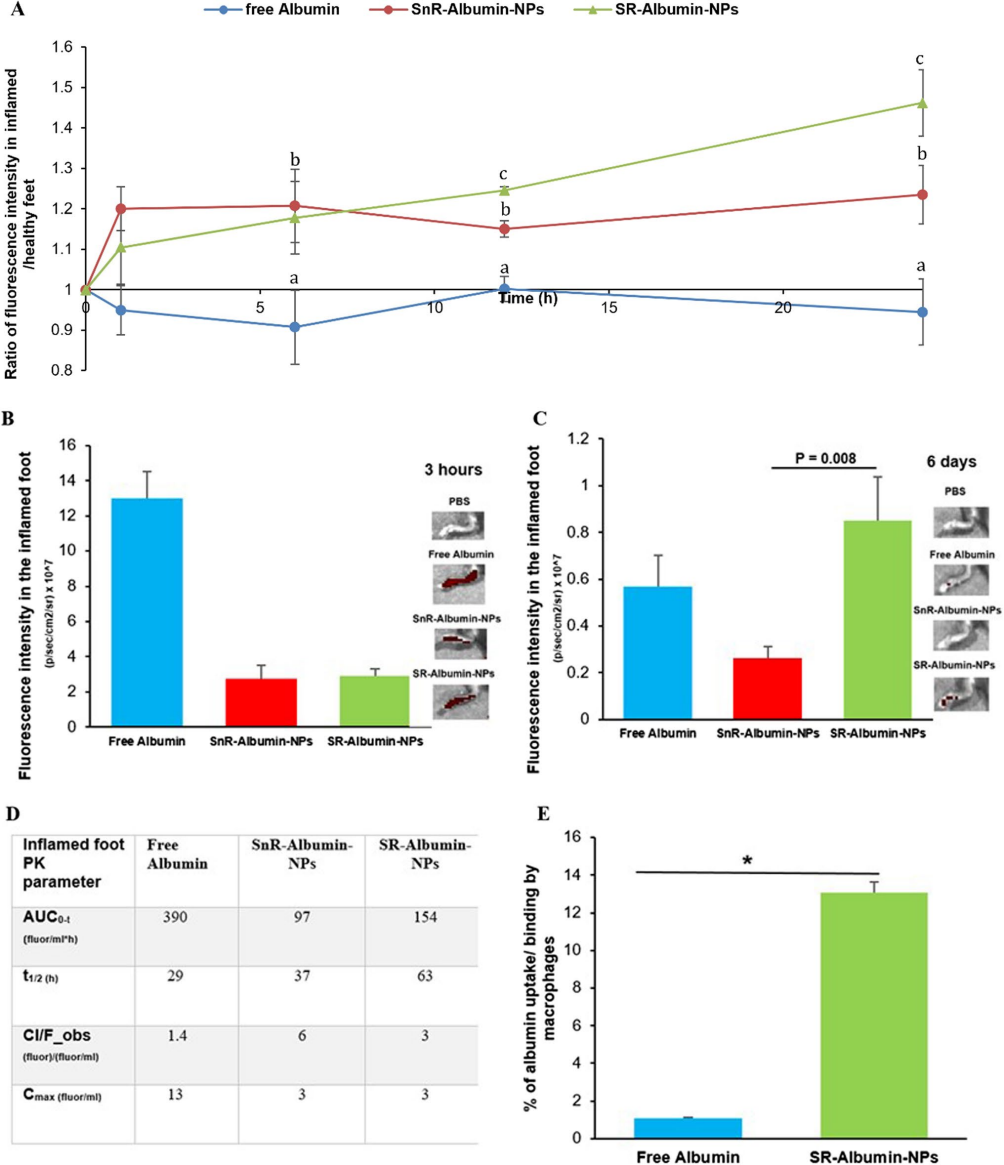
Selective distribution and retention of the free albumin, SnR-Albumin nanoparticles and the SR-Albumin nanoparticles in inflamed mouse foot. **A** In vivo specificity profile towards the inflamed foot of the free albumin, SnR-Albumin nanoparticles, and the SR-Albumin nanoparticles within 24 h post i.v. injection. A ratio of 1 indicates no difference in fluorescence between healthy and inflamed feet. **B** In vivo fluorescence intensity values measured in the inflamed foot at 3 h post i.v. injection, which is the Tmax. On the right are representative in vivo fluorescence images of the inflamed mouse feet at 3 h post i.v. injection. **C** In vivo fluorescence intensity values measured in the inflamed foot on day 6 post i.v. injection. On the right of the figure are representative in vivo fluorescence images of the inflamed mouse feet on day 6 post i.v. injection. **D** Selected PK parameters of SnR-Albumin nanoparticles and the SR-Albumin nanoparticles in the inflamed foot. Albumin from bovine serum (BSA) is conjugated to Alexa Fluor™ 680. **E** Uptake and/or binding of fluorescein-labeled albumin by J774A.1 macrophages. J774A.1 cells (2 × 10^5^) were seeded. Twenty hours later, the medium was replaced with serum-free DMEM containing fluorescein-labeled free albumin or SR-Albumin nanoparticles. The cells were washed after 50 min of incubation and lyzed, and the fluorescence intensity was measured. Data are mean ± S.E. (n = 3–5). (**A**–**C**, *p* < 0.05) or (*, *p* < 0.05)

Mice with LPS-induced chronic inflammation in the right hind footpad were i.v. injected with free BSA, the SnR-albumin nanoparticles, or the SR-albumin nanoparticles, all labeled fluorescently. As shown in Fig. 4A, both SnR-albumin nanoparticles and SR-albumin nanoparticles showed a higher distribution in the inflamed footpad relative to the healthy footpad, whereas the free BSA did not show selectivity toward the inflamed footpad. This finding is in agreement with the finding of the gold nanoparticles of various sizes as mentioned above (Figs. 1 and 2). Interestingly, 12 and 24 h after the injection, the SR-albumin nanoparticles also showed a relatively higher presence in the inflamed footpad than the SnR-albumin nanoparticles (Fig. 4A), likely due to the faster release of albumin from the SR-albumin nanoparticles (than from the SnR-albumin nanoparticles) in chronic inflammation sites, thereby enhancing its sequestration at the site of inflammation.

Shown in Figs. 4B–D are the fluorescence intensity values in the inflamed footpad of mice 3 h and 6 days after i.v. injection of BSA or albumin-nanoparticles. Three hours after the injection, the fluorescence intensity in the inflamed foot reached the highest (i.e., t_max_ = 3 h, data not shown). As expected, the fluorescence intensity in the inflamed footpad in mice injected with the BSA was significantly higher than in mice injected with the nanoparticles, and there was not any significant difference between mice injected with the SR-albumin nanoparticles and the SnR albumin-nanoparticles (Fig. 4B). However, 6 days after the injection, the fluorescence intensity in the footpad in mice i.v. injected with the SR-albumin nanoparticles was higher than that in mice i.v. injected with free BSA, whereas the fluorescence intensity in the inflamed footpad in mice injected with the SnR-albumin nanoparticles was the lowest (Fig. 4C), indicating that the albumin in the SR-albumin-nanoparticles had relatively the highest retention in the chronic inflammation sites. It is speculated that the increased retention of albumin in the SR-albumin-nanoparticles in chronic inflammation sites was related to the impaired lymphatic drainage during lymphangiogenesis in chronic inflammation sites [32]. The “zipper pattern” is rapidly formed in the lymphatics during inflammation and is known to reduce the permeability of fluid and access of smaller particles to the lumen of the lymphatics, thereby reducing smaller particles clearance [32, 47]. However, immune cell sequestration and trafficking (e.g., lymph node macrophages movement in and out of inflammation sites) is highly activated between the lymph node and inflamed tissues [32, 47]. It is known that smaller nanoparticles have fewer ligand-to-receptor interactions than larger ones, and thus they are less efficient in triggering cellular uptake via membrane wrapping [48, 49]. On the other hand, large nanoparticles (e.g., 200 nm) can attract receptor binding to successfully trigger uptake by the immune cells [48, 49]. We actually studied the uptake/binding of free albumin or albumin nanoparticles by J774A.1 macrophages in culture and found that the uptake/binding of the SR-albumin nanoparticles by the macrophages was significantly higher than free albumin (Fig. 4E). Nanoparticles size and charge are known to affect cellular uptake by inflammatory cells [50]. Therefore, the highly activated immune cell sequestration and trafficking of macrophages via the lymph nodes may have contributed to the faster clearance of the large SnR-albumin nanoparticles from the chronic inflammation sites. Free albumin and albumin released from the SR-albumin nanoparticles were not as readily taken up by immune cells and thus remained in the inflammation sites, which supports our hypothesis that nanoparticles of 100–200 nm that disintegrate into small nanoparticles of 2–10 nm in chronic inflammation sites would have a more selective distribution to the chronic inflammation sites than small nanoparticles of 2–10 nm and enhanced retention in the chronic inflammation sites than nanoparticles of 100–200 nm that do not disintegrate into small nanoparticles in chronic inflammation sites.

### SR-IgG-NPs enhanced the distribution and retention of IgG in chronic inflammation sites

**Fig. 5:**
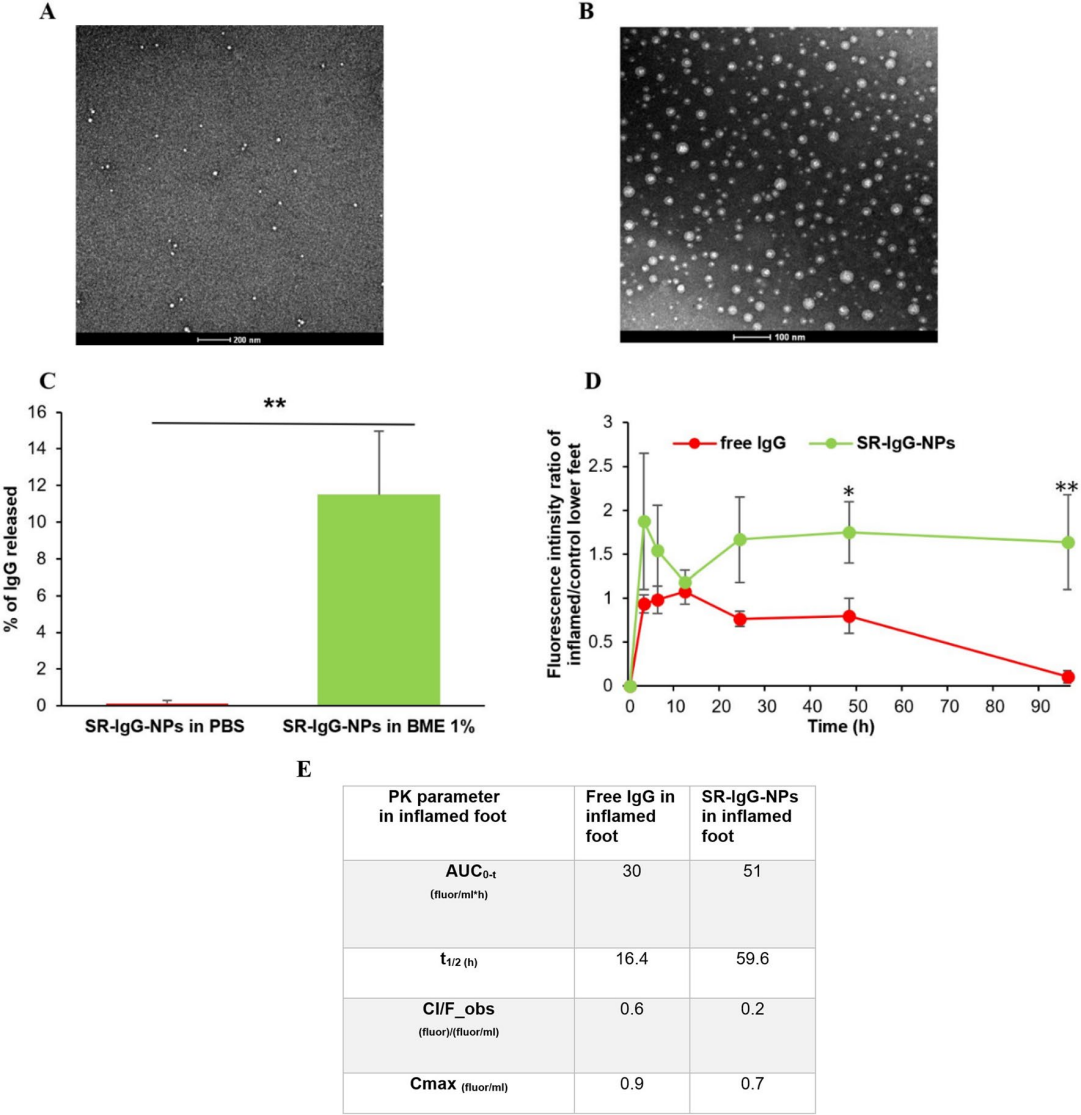
Distribution of IgG and SR-IgG-NPs in inflamed foot relative to healthy foot in the same mouse with chronic inflammation. **A** A representative TEM image of the free IgG. **B** a representative TEM image of the SR-IgG-NPs. **C** In vitro release of SR-IgG-NPs 2 h after pre-incubation in PBS or 1% of 2-mercaptoethanol. **D** Free IgG and SR-IgG-NPs fluorescence intensity profile in the inflamed feet of the mice. **E** Selected PK parameters of free IgG and SR-IgG-NPs in the inflamed feet of mice. Data are mean ± S.E. (n = 4). (*p ≤ 0.05, **p ≤ 0.01)

Monoclonal antibodies (mAbs) such as anti-TNF-α mAbs are used extensively in clinics to treat chronic inflammatory diseases, but are associated with adverse effects, likely due to their systemical neutralization of key cytokines [33–36]. We therefore tested whether crosslinking IgG into redox-sensitive nanoparticles will increase the selective distribution of the IgG toward chronic inflammation sites, relative to healthy tissues. The SR-IgG-NPs synthesized were 60–80 nm in size based on TEM images (Fig. 5A, B) and showed redox-sensitivity to 2-mercaptoethanol in vitro (Fig. 5C).

Figure 5D shows the PK profiles of free IgG and SR-IgG-nanoparticles in inflamed mouse footpad after i.v. injection and Fig. 5E contains selected PK parameters of IgG and SR-IgG-NPs in the inflamed footpad. The SR-IgG-NPs showed a significantly higher AUC in the inflamed footpad than the free IgG, which is known to lack specificity toward the inflamed footpad. Overall, formulating IgG into redox- and -pH sensitive nanoparticles of ~ 100–200 nm can increase its distribution and retention in chronic inflammation sites.

**Fig. 6:**
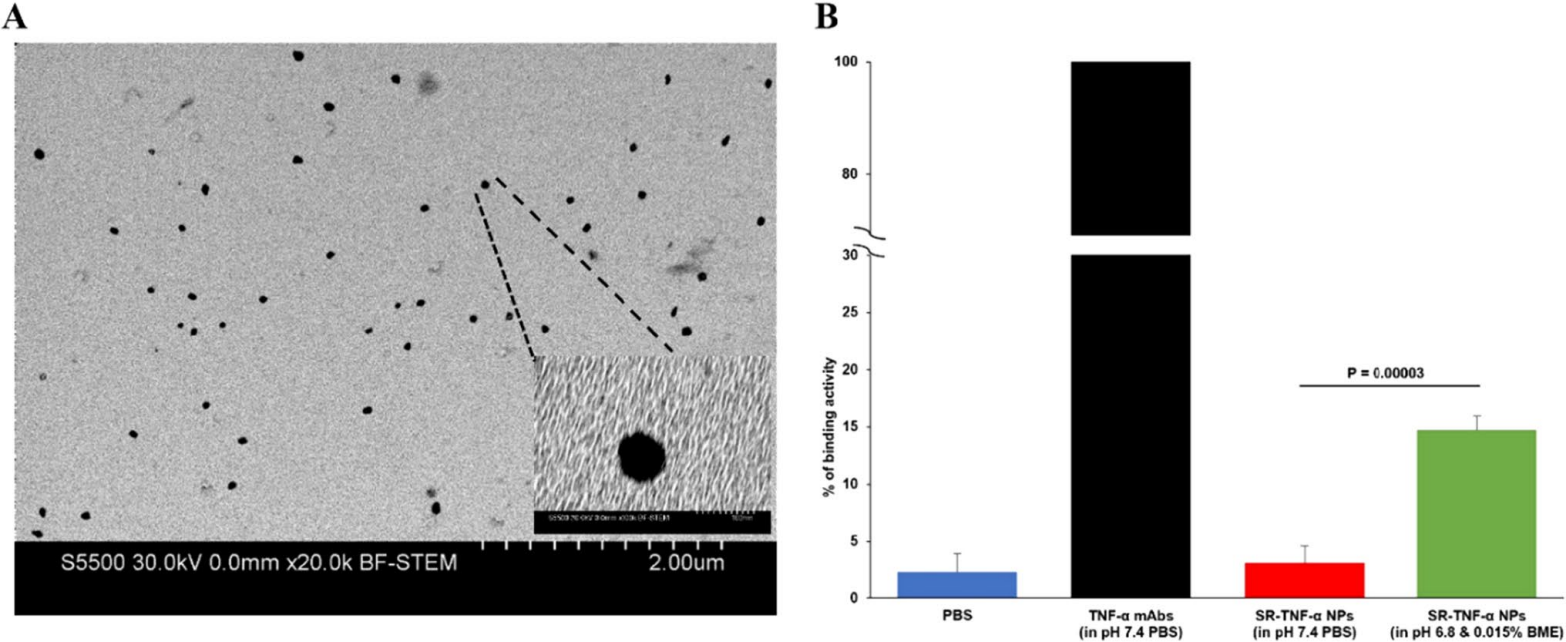
TNF-α mAbs in the SR-TNF-α mAb nanoparticles are still effective in binding to mouse TNF-α protein. **A** A representative STEM image of the SR-TNF-α-NPs. **B** The binding ability of TNF-α mAbs in the SR-TNF-α mAb nanoparticles were measured by ELISA. The binding ability of the SR-TNF-α mAb nanoparticles is dependent on higher redox activities provided by low pH (i.e., pH 6.8) and a reducing agent such as beta-mercaptoethanol (i.e., 0.015% BME). Data are mean ± S.E. (n = 3)

To test whether the mAbs released from the SR-IgG-nanoparticles in a high redox condition are still functional (i.e., can bind to their antigens), we prepared SR-anti-TNF-α-IgG-NPs using anti-mouse TNFα mAbs. The resultant nanoparticles are similar in size to SR-IgG-NPs (Fig. 6A). Importantly, as shown in Fig. 6B and Supp Fig. 1, the TNF-α mAbs released from the SR-TNF-α mAb nanoparticles in response to low pH (pH 6.8) and the presence of β-mercaptoethanol can still bind to TNF-α.

Taken together, we have shown that converting proteins such as mAbs into nanoparticles of 100–200 nm using disulfide bonds that are redox- and pH-sensitive can significantly increase the selective distribution of the proteins and extend their retention in chronic inflammation sites, while the proteins remained functional after they were released from the nanoparticles. This finding can be significant as most of the mAb therapies for chronic inflammation diseases, such as rheumatoid arthritis, are IgG-based. It is noted that this finding may only be applicable to mAbs that are given by intravenous injection or and infusion such as Infliximab [35, 51]. IgGs have increased localization at inflammation sites primarily due to their influx to the expanded extracellular space. However, IgGs freely distribute to healthy tissues as well. Packaging mAbs into 100–200 nm nanoparticles such as the SR-IgG-nanoparticles is expected to increase their selectivity toward chronic inflammation sites as well as their retention in chronic inflammation sites, potentially reducing their unwanted offsite activity and toxicity. Of course, the mAbs need to be released efficiently from the nanoparticles and remain functional.

In the present study, the proteins, albumin, IgG, or anti-TNF-α mAbs, were converted to nanoparticles of about 100–200 nm using a desolvation technique followed by crosslinking with disulfide bonds. The resultant nanoparticles released the proteins in response to high redox and/or low pH conditions, and the anti-TNF-α mAbs released can bond to TNF-α. It is noted that the method of transforming the proteins to stimuli-sensitive nanoparticles may need further optimization as proteins such as mAbs have their own disulfide bonds. However, the proof-of-concept findings shown in the present study demonstrated that it is feasible to increase the distribution and retention of protein therapeutics in chronic inflammation tissues, relative to healthy tissues, by formulating them into stimuli-sensitive nanoparticles of 100–200 nm in diameter. It is noted that in the present study, IVIS imaging was applied to determine the fluorescence intensity of various nanoparticles in mouse footpad, and the fluorescence intensity values were then used to understand the PK and distribution of the nanoparticles. Although similar method has been used by others in PK and biodistribution studies in animal models [17, 18, 22], and the nanoparticles used in the present studies were stably labeled with the fluorescent molecules, there are limitations associated with this method [37–39]. In addition, not all mAbs are administered by the intravenous route, and the findings in the present studies may not be applicable to nanoparticles and mAbs administered via other routes.

## Conclusion

In the present study, we showed that the size of nanoparticles significantly affected their selectivity, distribution, and retention in chronic inflammation sites, with small 2 nm and 10 nm showing higher distribution and longer retention in chronic inflammation sites than large 100 and 200 nm particles, but the large 100 nm and 200 nm nanoparticles are more selectively distributed to inflammation sites than the small 2 nm and 10 nm nanoparticles, which distributed equally to inflammation sites and healthy tissues. We then presented a strategy to increase the selective distribution of nanomedicines or proteins to chronic inflammation sites and prolong their retention in the sites by engineering nanoparticles that are sensitive to high redox activity and/or low pH, conditions seen in chronic inflammation sites.

### Supplementary Information

**Supplementary material**

## Data Availability

The datasets generated during and/or analyzed during the current study are available from the corresponding authors upon reasonable request.
