# Supplementary material for: Effect of nanoparticle size on their distribution and retention in chronic inflammation sites
Source: Discov Nano. 2023 Aug 22;18(1):105. doi: 10.1186/s11671-023-03882-w (PMC10444937; doi:10.1186/s11671-023-03882-w)
Supplement: Supplementary file 1 — Supplementary material [file 11671_2023_3882_MOESM1_ESM.docx]

**Supplementary Tables and Figures**

**Supp Table 1.** Selected PK parameters of 2 nm, 10 nm, 100 nm, and 200 nm fluorescently labeled gold nanoparticles in the healthy feet in mice.

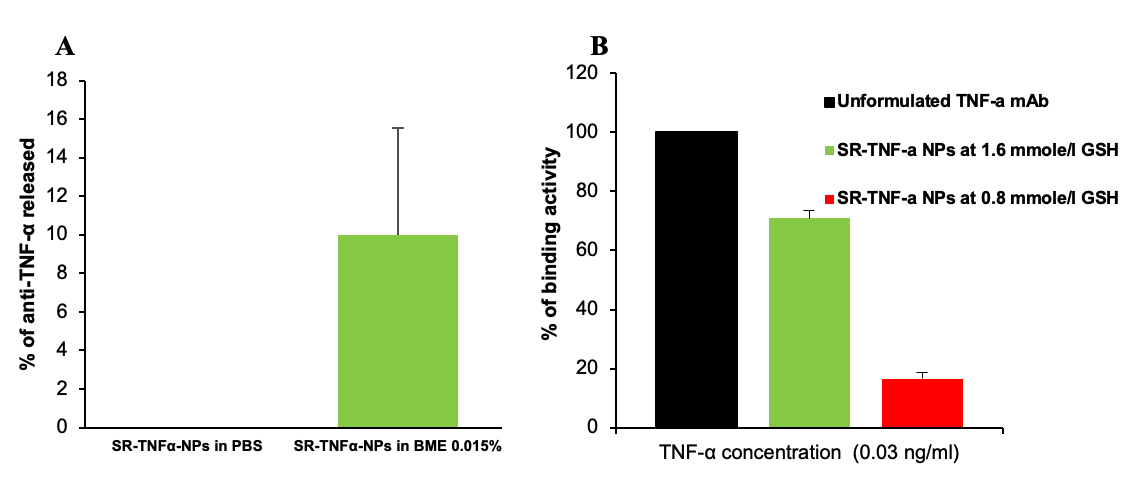


**Supp Fig. 1.** (A) In vitro release of anti-TNF-α mAbs from the SR-TNF-α mAb nanoparticles after 2 h of incubation in PBS or 0.015% of 2-mercaptoethanol. (B) The TNF-α binding ability of the anti-TNF-α mAbs in the SR-TNF-α mAb nanoparticles was dependent on the concentration of the reducing agent GSH (i.e., 1.6 vs. 0.8 nmole/ml).
